# From photoperiod thresholds to photoperiod sensitivity: dual strategies for cost-effective speed breeding and climate-ready barley

**DOI:** 10.3389/fpls.2026.1742787

**Published:** 2026-02-10

**Authors:** Nicola Rossi, Wayne Powell, Karen Halliday, Rajiv Sharma

**Affiliations:** 1 Scotland’s Rural College (SRUC), Edinburgh, United Kingdom; 2 Institute of Molecular Plant Sciences, School of Biological Sciences, University of Edinburgh, Edinburgh, United Kingdom

**Keywords:** climate-resilient varieties, energy savings, Hordeum vulgare L (Barley), response to photoperiod, speed breeding

## Abstract

**Introduction:**

Speed breeding (SB), characterized by extended photoperiods to accelerate generation time, can be energy-intensive, and the minimum day length required to trigger rapid flowering remains unknown. Additionally, climate change raises the need for shorter growing seasons in certain European regions, and reducing the time to flowering could be an effective strategy to mitigate its effects. Therefore, exploring how allelic combinations shape flowering time is needed. We present the first integrated study of how allelic variation at three key flowering time genes —*PPD-H1*, *ELF3*, and *PHYC* — modulates three parameters of the photoperiod response model: threshold photoperiod, photoperiod sensitivity, and intrinsic earliness.

**Materials and methods:**

We recorded flowering under lengths of 16–24h in Near Isogenic Lines carrying *PhyC-e* or *PhyC-I* allele within *ppd-H1* background, and in lines from HEB-25 combining wild and domesticated alleles of *ELF3* and *PPD-H1*.

**Results and discussion:**

Remarkably, *ppd-H1* lines flowered at a 20-h threshold, whereas *Ppd-H1* lines showed no response, consequently we propose new SB photoperiods at 20 and 16h depending on *PPD-H1* background. These photoperiods lower energy costs compared to the current 22h standard. In addition, the wild *ELF3* allele in *ppd-H1* background reduced intrinsic earliness, whereas *PhyC-e* reduced photoperiod sensitivity, opening opportunities for climate change adaptation.

## Introduction

Flowering time under very long photoperiods is an important consideration in speed breeding, which aims to shorten generation time (Hickey et al., 2017; Watson et al., 2018; Chiurugwi et al., 2019; Cazzola et al., 2020; Mobini et al., 2020; Samineni et al., 2020; Fang et al., 2021; Schilling et al., 2023). According to the breeder’s equation (Lush, 1937), genetic gain per unit time can be increased not only by applying selection but also by reducing generation time. The ability of crop plants to respond to increasing day lengths in controlled environments is instrumental in refining protocols that accelerate generation turnover, thereby enhancing genetic gain (Cobb et al., 2019).

Speed breeding protocols manipulate conditions like photoperiod and temperature to enable multiple generations per year. For long day plants (LDP) such as barley, wheat, canola, chickpea, pea and oat, speed breeding protocols typically employ a 22-hour photoperiod combined with a 2-hour dark phase and cooler night temperatures to accelerate generation turnover (Watson et al., 2018; González-Barrios et al., 2021). The 2-hour dark period, alongside reduced night temperatures, facilitates plant recovery and minimizes stress linked to accelerated growth (Watson, 2019). However, this approach may overlook key findings from photoperiod response modelling in long-day plants (Major, 1980) and has limitations when diverse germplasm (e.g. wild relatives) is used (Rossi et al., 2024). Experiments investigating major flowering time genes under defined photoperiod conditions have enhanced our understanding of the genetic control of flowering, informing strategies to improve adaptation in breeding programs (Pérez-Gianmarco et al., 2019).

In cereal crops, flowering time is a crucial trait for both adaptation (Jones et al., 2008; Faure et al., 2012) and for optimizing potential yield (Wang et al., 2009; Mäkinen et al., 2018), as these factors are often closely linked (Slafer et al., 2023). Climate change leads to less farmland and lower crop production (Asseng et al., 2015; Challinor et al., 2014) in key agricultural areas due to irregular and extreme weather conditions during the growing season (Hatfield et al., 2014). This erratic climate can lead to a shortening of the crop season. Longer crop seasons allow more time for plants to capture resources and grow, which can increase total yield (Guarin et al., 2022; Sinclair and Jamieson, 2006). In fact, areas with longer cropping seasons tend to produce higher yields (Magrin et al., 1993; Semenov and Shewry, 2011). Hot conditions during critical stages of crop growth can sharply reduce productivity. To counter these climate-driven risks, breeders require precise toolkits to select allele combinations optimized for specific production environments.

Recent studies show that the best areas for important crops are moving further north (Heikonen et al., 2025; Minoli et al., 2022; Sloat et al., 2020; Tan et al., 2021). In fact, northward shift of warmer climate (Rutkoski et al., 2016) might create an opportunity for a re-evaluation of the currently unexploited areas in the boreal region as to their suitability for agriculture (Zabel et al., 2014), regions where arable agriculture is currently not considered feasible (King et al., 2018). This raises the need to study the response of crops to longer photoperiod conditions than the usually assumed for long day conditions (i.e. 16 hours) to create germplasm that can adapt to these new photoperiod regimes.

This depth of understanding presents a strategic opportunity: by targeting photoperiod-responsive genes within a highly isogenic background, we can more precisely investigate how extended photoperiods affect flowering time— refining speed breeding protocols, advancing our understanding of adaptation and informing breeding decisions.

Among genetic factors, flowering time in small-grain cereals such as wheat and barley is governed by genotype and genotype-by-environment interactions, with the photoperiod and vernalization pathways serving as central regulators of day-length and low-temperature responses, respectively. In barley broad adaptation arises from extensive allelic variation within these pathways (Fernández-Calleja et al., 2021), which distinguishes winter and spring growth habits. Spring barley genotypes exhibit null or reduced vernalization requirements, relying predominantly on photoperiod sensitivity to delay flowering under long days. Once photoperiod and vernalization responses are saturated, residual genotypic variation in flowering time is mainly controlled by earliness per se (eps) loci (Slafer and Rawson, 1994; Parrado et al., 2023).

In barley (Hordeum vulgare L.), flowering time exhibits a characteristic bi-linear response to photoperiod duration. The seminal work by Major (1980), in relation to the photoperiodic response, established that small grain cereals display: (i) an initial linear decline in flowering time with increasing photoperiod until reaching a threshold (*threshold photoperiod*), beyond which (ii) daylength extension no further accelerates development. The slope of the initial linear phase (*photoperiod sensitivity*) quantifies the responsiveness to daylength, while the stable phase represents the degree of *intrinsic earliness* - the minimum flowering time when photoperiod requirements are fully satisfied. Such a parameter is known to be controlled by eps genes, such as barley *CENTRORADIALIS* (*CEN*) (Fernández-Calleja et al., 2021). In barley and other temperate cereals, key genes such as wheat *PHOTOPERIOD1 (PPD1)* and its barley orthologue *PPD-H1*, as well as earliness per se genes, underpin variation in these photoperiodic traits. Pérez-Gianmarco et al. (2019) applied Major’s (1980) photoperiod response model to characterize *PPD1* alleles in wheat (*Triticum aestivum* L.), revealing conserved threshold photoperiod and intrinsic earliness across genotypes, with variation occurring primarily in photoperiod sensitivity. In barley (*Hordeum vulgare* L.), Parrado et al. (2023) conducted complementary studies using near-isogenic lines (NILs) for *PPD-H1* across controlled and field environments. While their study did not employ formal modelling, the results demonstrated that *PPD-H1* alleles modulate both photoperiod threshold and sensitivity, while maintaining stable intrinsic earliness.

Photoperiodic flowering in grasses is regulated by a network of genes that coordinate environmental signals with developmental responses. Central to this network is the gene *PPD1*, which acts as a molecular switch to initiate flowering in response to day length (Turner et al., 2005). *PPD1* encodes a pseudo-response regulator protein (*PRR37*) that promotes the expression of *FLOWERING LOCUS T1* (*FT1*), the key integrator of flowering signals, leading to floral transition under long days (Shaw et al., 2020). The regulation of *PPD1* expression results from the interplay between the circadian clock and light signaling pathways, such that its activation occurs when the timing of gene expression coincides with periods of light under long-day conditions (Song et al., 2015). This mechanism ensures that *PPD1* is only activated when internal circadian rhythms align with specific external cues, such as light. Upstream regulators of *PPD1* include the circadian clock component *EARLY FLOWERING 3* (*ELF3*) and light-sensing phytochromes, especially *PHYTOCHROME C (PHYC)*. In model grasses such as *Brachypodium*, *PHYC* has been shown to likely repress *ELF3* post-translationally (Bouché et al., 2022; Alvarez et al., 2023; Gao et al., 2023). As a result, *ELF3* cannot suppress *PPD1*, leading to the flowering response via the induction of *FT1* expression. However, the precise molecular interactions among these clock components in barley are less well characterized, and much of our current understanding is extrapolated from these related grass (*Brachypodium*, wheat and rice) systems. Nevertheless, research in barley has confirmed the roles of functional allelic variation at *PHYC*, *PPD-H1* (*PPD1* orthologue) and *ELF3* in modulating photoperiod sensitivity and flowering time (summarized below).

The effects of variation at *PPD-H1* indicate the presence of two functional alleles: the dominant wild allele *Ppd-H1*, which causes early flowering phenotypes, and the recessive *ppd-H1*, which harbors a mutation within the CCT domain. This mutation is thought to reduce its ability to activate *FT1* compared to *Ppd-H1* under long-day conditions (Sharma et al., 2025; Turner et al., 2005), leading to a delay in flowering. The recessive mutation giving rise to *ppd-H1* favored the expansion of barley from the Fertile Crescent to higher latitudes (von Bothmer and Komatsuda, 2010), characterized by longer growing seasons. Therefore, *ppd-H1* is preferred in regions characterized by long growing seasons (such as central and northern Europe) and *Ppd-H1* in environments characterized by higher temperatures and drought (e.g. the Mediterranean basin) (Wiegmann et al., 2019). The *ELF3* allelic series comprises three main alleles: the domesticated allele *Elf3*, the *elf3* alleles, and the wild *ELF3_Hsp_*found in *Hordeum* sp*ontaneum* lines. The *elf3* alleles comprise two alleles, the *eam8.k* which contains two deletions, one inversion, and two small insertions and *eam8.w* allele which has a point mutation that causes a premature stop codon (Faure et al., 2012; Zakhrabekova et al., 2012; Zahn et al., 2023). These mutations result in photoperiod insensitivity and early flowering both in long and short days, likely due to lack of repression of *PPD-H1*, which enhances *FT1* activation and disrupts the circadian clock (Müller et al., 2020). *elf3* alleles have been recognized as a crucial factor aiding barley’s adaptation to very short growing seasons at high latitudes (Faure et al., 2012). It has been proposed that the wild *ELF3_Hsp_* allele is thought to contain a non-synonymous mutation at amino acid position 669, contributing to an acceleration of flowering (Zahn et al., 2023). Allelic series at barley’s *PHYC* gene involve the wild *PhyC-I* and the *PhyC-e* allele that harbors a mutation in a critical position within the GAF domain, located at the end of a helix near the chromophore pocket. This mutation causes a notable reduction in flowering time. *PhyC-e* is thought to bypass the circadian clock genes inducing *PPD-H1* in barley which then leads to an enhanced accumulation of *FT1* and early flowering (Nishida et al., 2013; Pankin et al., 2014).

The importance of *ppd-H1*, which contributes to extending the growing season at moderately long days, may decline in some central European regions as temperatures continue to rise. It has been suggested that *Ppd-H1* could play a more prominent role under irregular and extreme weather conditions during the growing season (Herzig et al., 2018), given its strong effect on accelerating flowering. However, relying solely on *Ppd-H1* may not always offer the optimal balance for adaptation. This emphasizes the importance of investigating allelic combinations in a *ppd-H1* background that support intermediate flowering times—providing greater flexibility for adapting to warmer climates without excessively shortening the growing season. The effect of *ELF3_Hsp_* and *PhyC-e* in a *ppd-H1* background may create intermediate phenotypes. These phenotypes could shorten the growing season enough to avoid terminal heat and drought caused by rising temperatures. However, they would still ensure a longer growing season than what *Ppd-H1* can provide. In summary, achieving stable yields in Central and Northern European agriculture requires a dual strategy: advancing flowering dates to mitigate climate-induced stress and preserving a growth duration long enough to support productivity. Furthermore, the poleward shift of agriculture necessitates an understanding of genetically mediated barley responses under very long days. This can help better act on allelic combinations that guarantee a variability to select from.

A recent study by Rossi et al. (2024) demonstrated that *ELF3* and *PPD-H1* are key regulators of developmental timing under both standard (i.e. 16 hours) and speed breeding (i.e. 22 hours) photoperiods. Notably, this revealed that domesticated alleles benefit the most in accelerating the growing cycle under speed breeding conditions. These findings highlighted that the effectiveness of speed breeding protocols is highly influenced by allelic variation, particularly within diverse germplasm pools. However, the photoperiod threshold required to trigger accelerated development—and its interaction with genotype—remains unexplored, posing a key limitation to the design of efficient and energy-smart speed breeding protocols. Cutting energy could reduce lighting-related energy costs by approximately 4.54% each hour. To put these savings into perspective, Zhang et al. (2017) reported that, in a greenhouse tomato case study, a 650−W LED fixture covering 8 sq ft and operated 3,000 h per year (45,000 h over 15 years) consumed a total of 29,250 kWh to produce tomatoes. Following these assumptions, reducing LED lighting by one hour per day in commercial breeding facilities can save approximately 237 kWh annually per 8 sq. ft. These savings scale up in larger operations, illustrating how genotype-informed speed breeding protocols can make breeding programs both more sustainable and cost-effective.

This study builds upon our recent research (Rossi et al., 2024) on how wild and domesticated alleles of *ELF3* and *PPD-H1* from the Nested Associated Mapping population HEB-25 (Maurer et al., 2015) influence flowering time under different photoperiods. We also examined *PHYC* alleles (*PhyC-e*, *PhyC-I*) using Bowman introgression lines (Druka et al., 2011). The photoperiods tested ranged from 16 to 24 hours. By modelling the photoperiod response in this range, we seek to determine the *threshold photoperiod* parameter that can be used to optimize energy-efficient speed breeding. Additionally, we aim to inform breeding of climate-resilient barley for central–northern Europe, where photoperiods are long to very long, by quantifying the allelic combinations that drive *photoperiod sensitivity* and *intrinsic earliness*, the other two parameters of the model. By targeting intermediate flowering times for long-day environments, we can sustain growth rates during the plant’s most critical developmental stages, thereby maximizing yield potential (Carrera et al., 2024; Slafer et al., 2023).

## Materials and methods

### Plant material

In this study, we investigated the photoperiod response model in two genetically distinct plant groups. The first group, referred to as the “HEB group,” consists of four recombinant inbred lines (RILs) from a HEB-25 family of the multiparent nested associated mapping (NAM) population “Halle Wild Barley” (HEB-25). This population was created by crossing 25 wild barley parents—24 *Hordeum vulgare* ssp. *spontaneum* (Hsp) and one *Hordeum vulgare* ssp. *agriocrithon*—with the spring barley cultivar Barke (*H. vulgare* ssp. *vulgare*, Hv). Barke is a European spring barley cultivar that has been widely cultivated in Northern Europe (CPVO, 2025). Its inclusion offers an agronomically relevant genetic background for assessing allelic variation at PPD-H1 and ELF3 under long-day conditions. The resulting progeny were backcrossed to the female elite barley variety Barke, followed by three generations of selfing through single-seed descent (BC_1_S_3_), and further propagated minimum to the BC_1_S_6_ generation. More details about the population development is given in Maurer et al. (2015). The HEB group includes the four possible allele combinations at the *ELF3* and *PPD-H1* loci. To ensure the most isogenic background possible available, these combinations were identified based on the lack of segregation at markers linked to four key flowering time genes (*CEN*, *SDW1*, *VRN-H1/PHYC*, and *FT1*), as determined using the Infinium iSelect 50k SNP array (Maurer and Pillen, 2019). The selected markers associated with the target flowering time genes were chosen based on the subset used for the HIF pre-selection in Zahn et al. (2023), the markers composition is provided in **Supplementary Table S1**. In the HEB group we designate the alleles as follows: the dominant *Ppd-H1* as wild *PPD-H1_Hsp_*, the recessive *ppd-H1* as domesticated *PPD-H1_Hv_*, the domesticated *Elf3* as *ELF3_Hv_* and the wild *ELF3_Hsp_*. Consequently, the factorial combination of *ELF3* and *PPD-H1* includes four lines: *ELF3*_Hv_/*PPD-H1*_Hv_, *ELF3*_Hv_/*PPD-H1*_Hsp_, *ELF3*_Hsp_/*PPD-H1*_Hv_, and *ELF3*_Hsp_/*PPD-H1*_Hsp_, these are also the names with which the lines are referred to (**Table 1**). However, for the latter combination, no genotype was found without segregation at the *FT1* locus. Despite this limitation, we included the closest available genotype as a representative of the *ELF3_Hsp_/PPD-H1_Hsp_* combination.

**Table 1:** Genotypic combinations of *ELF3* and *PPD-H1* alleles in the HEB group analyzed in this study.

Genotype	*ELF3* allele (origin)	*PPD-H1* allele (origin)
*ELF3*_Hv_/*PPD-H1*_Hv_	Domesticated (*H. vulgare*)	Domesticated (*H. vulgare*)
*ELF3*_Hv_/*PPD-H1*_Hsp_	Domesticated (*H. vulgare*)	Wild (*H.* sp*ontaneum*)
*ELF3*_Hsp_/*PPD-H1*_Hv_	Wild (*H.* sp*ontaneum*)	Domesticated (*H. vulgare*)
*ELF3*_Hsp_/*PPD-H1*_Hsp_	Wild (*H.* sp*ontaneum*)	Wild (*H.* sp*ontaneum*)

The alleles are derived from either the domesticated barley *Hordeum vulgare* (Hv) or the wild progenitor *Hordeum* sp*ontaneum* (Hsp) from HEB-25 population.

The second plant group, named “Bowman group” (Druka et al., 2011) consisted of the wild-type cultivar Bowman and its respective near-isogenic lines (NILs) the allele *elf3* (eam8.w from the line BW290, from Zakhrabekova et al., 2012) and *PhyC-e (*line name BW285, from Pankin et al., 2014). Bowman is an older North American spring barley cultivar adapted to late season heat and drought stress (Franckowiak et al., 1985). Although it is not representative of modern European cultivars, Bowman provides a genetically stable and well-characterized background in which the effects of individual loci, such as PHYC, can be examined with minimal background interference. In addition, its adaptability to drought stress provides a strong candidate background for future central-northern European TPEs (Target Population of Environments). The lines in this group are referred to as BW_WT_, BW_ELF3_, and BW_PHYC_, respectively (**Table 2**). The rationale for having the photoperiod insensitive line BW_ELF3_ is to have a line which will express *intrinsic earliness*.

**Table 2:** Genotypic composition of the Bowman introgression group, consisting of the wild-type cultivar Bowman (BW_WT_) and two near-isogenic lines (NILs) differing at the *ELF3* and *PHYC* loci: BW_ELF3_, carrying the *elf3* mutant allele (eam8.w), and BW_PHYC_, carrying the *PhyC-e* allele.

Genotype	*ELF3* allele	*PPD-H1* allele	*PHYC* allele
BW_WT_	*ELF3_Hsp_*	*ppd-H1*	*PhyC-I*
BW*_ELF3_*	*elf3*	*ppd-H1*	*PhyC-I*
BW_PHYC_	*ELF3_Hsp_*	*ppd-H1*	*PhyC-e*

All lines share the same recessive *ppd-H1* background and differ at either the *ELF3* or *PHYC* locus. Allele origins and mutations are based on Druka et al. (2011); Zakhrabekova et al. (2012), and Pankin et al. (2014).

To ensure that the RILs used in the HEB group harbored the different *ELF3* and *PPD-H1* alleles, we sequenced these genomic regions and compared them with Barke and Bowman. Graphical genotyping is provided in Data S1 along with full details of DNA extraction, amplification, and sequencing procedures.

### Experimental design and phenotyping

To ensure precise photoperiod control and minimize light leakage, plants were grown in 80×80×160 cm grow tents (Senua Hydroponics-https://www.senua-hydroponics.com/) under strictly regulated conditions. An illustration of the experimental setup is provided in **Supplementary Figure S1**. Seeds were sown directly in 0.3 liter pots with Sinclair All Purpose Growing Medium Compost (https://www.sinclairpro.com/). Plants were exposed to 5 photoperiod conditions: 1) 16 h light/8 h darkness, 2) 18 h light/6 h darkness, 3) 20 h light/4 h darkness, 4) 22 h light/2 h darkness and 5) 24 h light/0 h darkness (continuous light). The experiment was repeated twice under identical conditions during the spring seasons (March to May) in SRUC’s Peter-Wilson campus (55°55′17.386″ N−3°10′42.175″ E) growth chambers in year 2023 and 2024. Five replicates of each line (7 lines in total) in each condition for each experiment repetition were grown in a completely randomized block design (RBD) within each tent. With 35 pots occupying the 0.64 m² tent space, this resulted in a planting density of 55 plants/m². The light intensity was set at 200 μmol m^-^² s^-^¹ as found to be optimal for promoting robust growth in barley (Yang et al., 2024). Light intensity was measured using a quantum sensor (SKP 200—Skye Instruments https://www.campbellsci.co.uk/skp215), while temperature was recorded every 30 minutes using dataloggers (EasyLog USB www.lascarelectronics.com). Temperature was set at 20°C constant.

Our study concentrated on flowering time response to different photoperiod conditions. The phase duration from emergence to when the awns become visible, defined as heading (BBCH stage 49, Lancashire et al., 1991) was used to determine time to flowering, which was expressed in accumulated thermal time to heading (flowering), assuming a base temperature of 0°C as described in Parrado et al. (2023).

### Photoperiod response modelling

Phenotypic data were manually curated to identify and remove erroneous measurements and biological outliers prior to analysis. Genotype means were visualized across photoperiod conditions, guided by the conceptual framework of Perez-Gianmarco et al. (2019), to select appropriate modelling strategies. This prior knowledge, combined with visual assessment of the data, informed whether:

- multiple lines could be modelled together while keeping some (or all) photoperiod-response parameters stable, or
- the model required inclusion of all parameters (photoperiod sensitivity, threshold photoperiod, and intrinsic earliness) to capture the photoperiod response. All modelling was performed in RStudio v2.14.4 using the *brms* R-package (Bürkner, 2017).

### Gene expression analysis

To assess whether the observed phenotypic variation could be attributed to the gene of interest rather than background genetic effects in the HEB group, we examined transcript abundance of candidate genes *PPD-H1* and downstream *FT1* in the four lines in the HEB group under contrasting photoperiods: 16h light/8h dark (16h; 20.9°C/16.4°C) and 22h light/2h dark (22h; 19.3°C/15.5°C).

Leaf samples were collected every 6 hours from ZT5 (Zeitgeber Time, indicating the hours after the onset of light) on day 23 post-emergence with two biological replicates and two technical replicates per line, photoperiod condition and time point. Following flash-freezing in liquid N_2_ and homogenization, total RNA was extracted using Qiagen’s RNeasy Plus Kit with QIAshredder. After DNase treatment (TURBO DNA-free™ Kit) and cDNA synthesis (SuperScript™ III), qPCR was performed in technical duplicate using SYBR^®^ Green chemistry on an AriaMx Real-Time System. *HvTubA* served as the reference gene, showing the most stable expression among tested references (*HvGAPDH*, *HvUbi*).Expression differences were assessed using two-sided t-tests. Primer sequences and cycling conditions are provided in **Supplementary Table S3**.

## Results

The results below integrate phenotypic modelling and gene expression analysis to reveal how genetic variation at flowering time loci may inform more energy-efficient speed breeding protocols and climate-resilient barley improvement.

### Photoperiod response models on thermal time to heading

**Figure 1:**
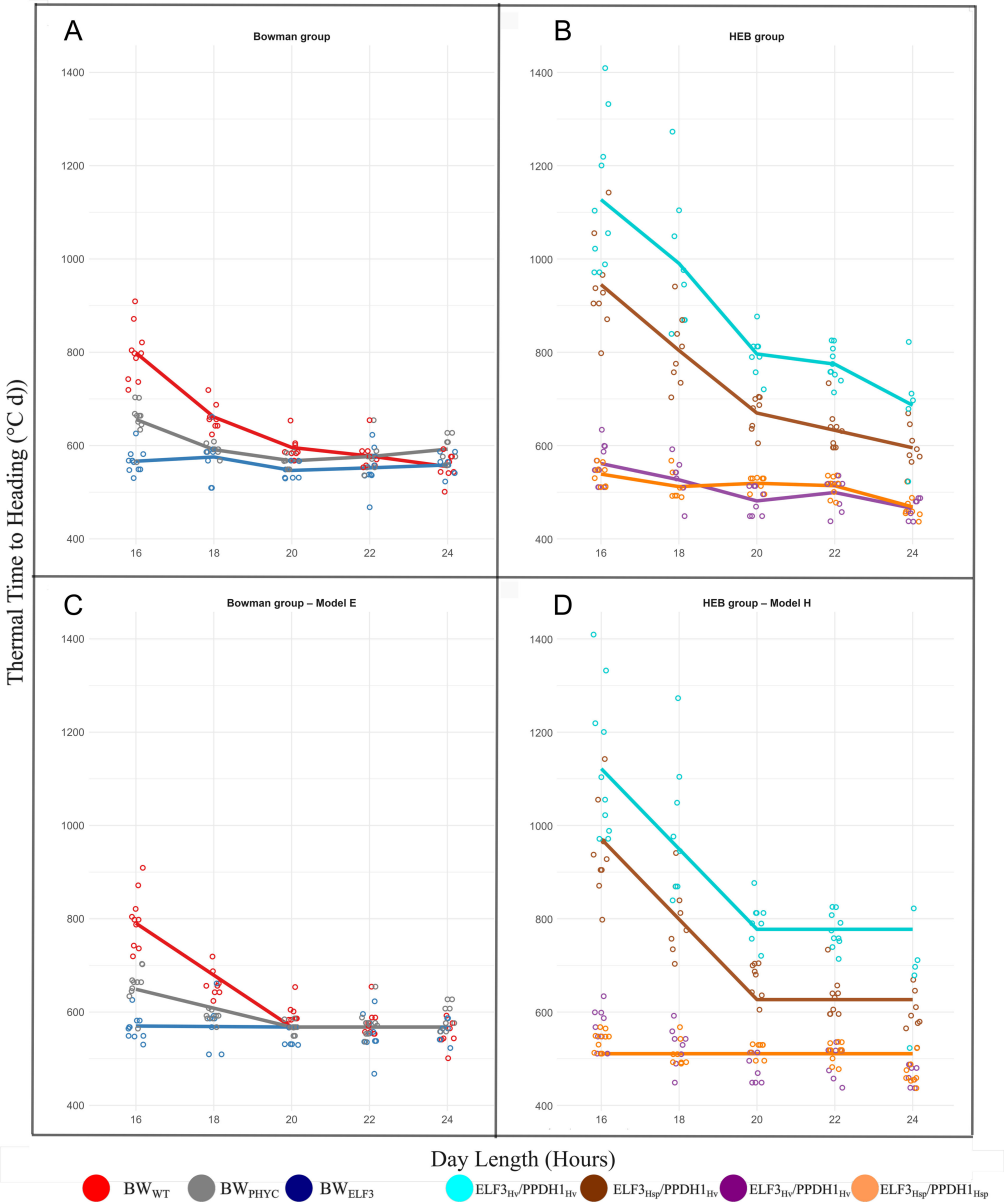
Flowering responses to increasing photoperiods in Bowman and HEB genotypes. Observed mean thermal time to heading for each genotype under five photoperiod conditions for Bowman group **(a)** and HEB group **(b)**;Fitted Bayesian model based on estimates of photoperiod sensitivity, threshold photoperiod, and intrinsic earliness for each line for Bowman group **(c)** and HEB group **(d)**.

To understand how genetic variation influences flowering responses under different daylengths, we measured the thermal time to heading across genotypes and modelled photoperiod responses. In the HEB group (**Table 1**), analysis of mean thermal time to heading for each line (**Figures 1a, b**) revealed that lines carrying the *PPD-H1*_Hv_ allele exhibited a bi-linear response with a distinct breakpoint at 20 hours. In contrast, lines with the *PPD-H1*_Hsp_ allele showed no response across the photoperiod range studied and consistently flowered earlier under all conditions. Within the *PPD-H1*_Hv_ background, lines with *ELF3*_Hsp_ flowered earlier than those with *ELF3*_Hv_, though both shared similar responsiveness up to the 20-hour breakpoint. In the Bowman group (**Table 2**), photoperiod sensitivity appeared to be modulated by allelic variation at *PHYC*. Although all genotypes converged to the same flowering time beyond 20 hours, they varied in intrinsic earliness beyond this threshold.

These empirical patterns informed our modelling strategy. We fitted Bayesian models (**Figures 1c, d**) to estimate the three key components of photoperiod response: photoperiod sensitivity, threshold photoperiod, and intrinsic earliness. This approach enabled to identify the specific effects of *PPD-H1*, *ELF3*, and *PHYC* alleles on the parameters defining our model equations. Detailed model specifications are provided in **Supplementary Data S2**.

Furthermore, pairwise Student’s t-tests were conducted to quantify differences in thermal time to heading between key photoperiod treatments (16 h vs 20 h and 20 h vs 22 h; **Supplementary Tables S2A, B**). All genotypes except BW_ELF3_ (as expected) exhibited significant reductions in thermal time to heading when the photoperiod was extended from 16 h to 20 h (p < 0.05), consistent with strong photoperiod sensitivity in this range. However, the magnitude of change was notably smaller in lines carrying the *PPD-H1*_Hsp_ allele compared to lines with photoperiod-responsive alleles (**Supplementary Figure S2**). When the photoperiod was further extended from 20 h to 22 h (the photoperiod used in speed breeding), did not result in statistically significant changes in thermal time to heading in any genotype, indicating convergence of flowering time above the 20 h threshold. These results delineate the photoperiod sensitivity window and highlighted the limited acceleration of *PPD-H1_Hsp_* lines relative to other genotype groups.

### Gene expression analysis

To further understand the phenotypic differences between HEB lines and photoperiod conditions, we carried out a gene expression analysis via RT-qPCR on *PPD-H1* and *FT1* grown at 16 and 22 h photoperiods. Most of the statistical differences in gene expression between lines were observed at 16h.

**Figure 2:**
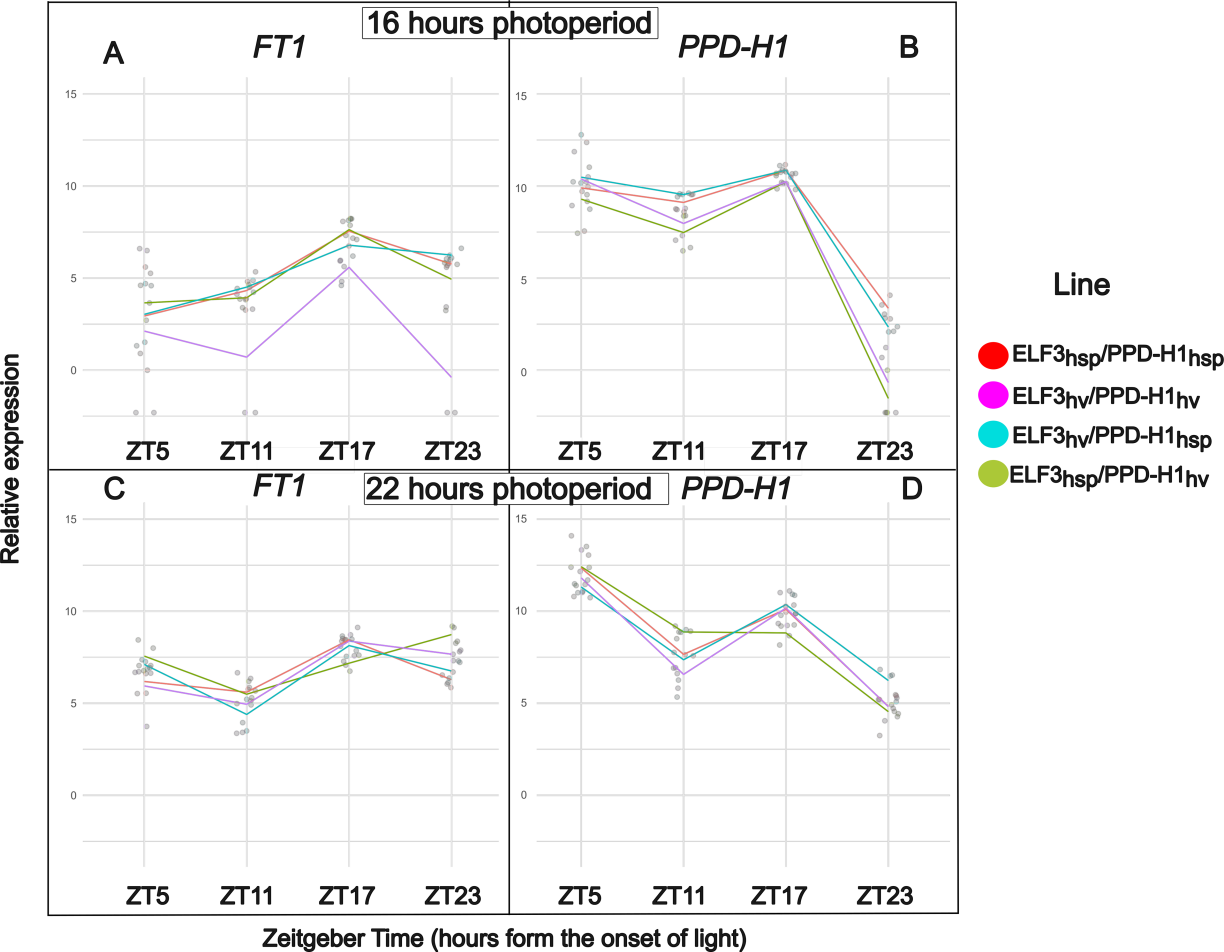
Expression of *PPD-H1* and *FT1* across photoperiods and time points in four genotypes. **(a)***FT1* under 16 h light/8 h dark at ZT5, ZT11, ZT17, and ZT23; **(b)***PPD-H1* under 16 h light/8 h dark at the same time points; **(c)***FT1* under 22 h light/2 h dark at ZT5, ZT11, ZT17, and ZT23; **(d)***PPD-H1* under 22 h light/2 h dark at the same time points. Points show means of two biological replicates (each with two technical replicates). Statistical comparisons of mean expression (two-sided Student’s t-tests) are reported in **Supplementary Tables S4** and **S5**.

*PPD-H1* expression was significantly higher in lines carrying the *PPD-H1_Hsp_* allele compared to those with *PPD-H1_Hv_* at ZT11, ZT17, and ZT23, under both 16 h (**Figure 2b**) and 22 h (**Figure 2d**) photoperiods (Student’s t-test, p-values in **Supplementary Table S4**). Notably, at ZT23—the only time point when lights were off in both photoperiod treatments, *PPD-H1_Hv_* expression was undetectable in four out of eight samples in the two lines. This correlates with the slower flowering time observed in lines harboring this allele. At ZT17, *PPD-H1* expression was also significantly higher in *ELF3_Hsp_*/*PPD-H1_Hv_* lines compared to *ELF3_Hv_*/*PPD-H1_Hv_*, correlating with the faster flowering time observed in the first line.

*FT1* expression was undetectable in *ELF3_Hv_*/*PPD-H1_Hv_* lines at ZT5, ZT11, and ZT23 under the 16 h photoperiod (**Figure 2a**), with expression absent in two out of four samples at each time point. As expected, statistical differences in *FT1* expression were primarily observed between genotypes differing at the *PPD-H1* locus, with higher expression in lines carrying the *PPD-H1_Hsp_* or *Ppd-H1* allele (Student’s *t*-test, *p*-values in **Supplementary Table S5**). The only exception was at ZT17 at 16h, where *ELF3_Hsp_*/*PPD-H1_Hv_* showed significantly higher expression than *ELF3_Hv_*/*PPD-H1_Hv_* despite sharing the same *PPD-H1* allele (**Figure 2a**). Such a result correlates with the faster flowering time observed in *ELF3_Hsp_*/*PPD-H1_Hv_*.

## Discussion

In this study, we build on our previous work (Rossi et al., 2024) by directly testing how different alleles of *PPD-H1*, *ELF3*, and *PHYC* (*Elf3* or *ELF3_Hv_*, *elf3*, *ELF3_Hsp_*, *ppd-H1* or *PPD-H1_Hv_*, *Ppd-H1* or *PPD-H1_Hsp_*, *PhyC-I*, and *PhyC-e*) affect key aspects of photoperiod response in barley under long days (16-18h) and very long days (above 18h) in controlled environments. To achieve this, both NILs in the Bowman background and RILs from the HEB-25 NAM population were used. The latter selected to minimize genetic variation in flowering time outside the target loci. This approach enabled the direct quantification of the effects of specific alleles on key aspects of the photoperiod response.

Our study is the first to assess how allelic variation at three major flowering time genes collectively influence threshold photoperiod, photoperiod sensitivity, and intrinsic earliness (Major, 1980; Perez-Gianmarco et al., 2019) within a single, unified experiment. By integrating flowering time data across a range of day lengths with gene expression analysis, we directly compared the effects of distinct allelic combinations at these loci on photoperiod response parameters. This comprehensive approach yields new insights into the genetic control of flowering in barley—specifically, by identifying the photoperiod threshold necessary to optimize energy-efficient speed breeding protocols (Watson et al., 2018; Hickey et al., 2019) and by elucidating patterns of photoperiod sensitivity and intrinsic earliness that will guide allele selection for adaptation to a warming climate (Slafer and Rawson, 1995; Craufurd and Wheeler, 2009; Reynolds et al., 2009).

Although our results revealed some statistically significant differences in thermal time to heading between long photoperiod treatments (such as 16 h compared to 20 h in lines harboring *Ppd-H1*), the magnitude of these differences was limited. Accordingly, we have focused our interpretation on the photoperiod response model, since it better captures the underlying biological mechanisms and supports robust comparisons across different genotypes and environments. Our findings show that lines carrying the *ppd-*H1 allele, including ELF3_Hv_/PPD-H1_Hv_, ELF3_Hsp_/PPD-H1_Hv_, BW_ELF3_ and BW_PHYC_—regardless of *ELF3* background—exhibited a clear bi-linear response to photoperiod, with flowering time accelerating below a threshold of 20 hours and then plateauing. In contrast, lines with the *Ppd-H1* allele, including ELF3_Hv_/PPD-H1_Hsp_ and ELF3_Hsp_/PPD-H1_Hsp_, showed no substantial response to increasing photoperiod and flowered early under all conditions, indicating that lines harboring this allele had already reached the threshold photoperiod at 16h. Consistent with the early-flowering phenotype, *PPD-H1* expression was significantly higher in lines carrying the *PPD-H1_Hsp_* allele compared to those with *PPD-H1_Hv_* under 16h. Such differences in expression correlate with levels of *FT1* expression with significant higher expression on lines carrying the *Ppd-H1*, In contrast to Parrado et al. (2023) different *PPD-H1* alleles yielded a different intrinsic earliness. This response correlated with a significant higher level of expression in *Ppd-H1* lines at *PPD-H1* and *FT1* in 22 h (after the threshold photoperiod). In addition, *ELF3_Hsp_* lines consistently headed earlier than *ELF3_Hv_*in a *ppd-H1* background, indicating that functional *ELF3* alleles primarily shift intrinsic earliness rather than threshold or sensitivity. This correlated with a significant higher expression of *FT1* in *ELF3_Hsp_*/*PPD-H1_Hv_* than *ELF3_Hv_*/*PPD-H1_Hv_*at ZT17 in 16h. Whereas the *PhyC-e* allele, harbored within the Bowman NIL *BW_PHYC_*, affected photoperiod sensitivity but not intrinsic earliness.

The data and observations obtained from this study provide valuable practical insights: the threshold photoperiod helps us understand how to optimize genotype-tailored speed breeding protocols. Current speed breeding approaches for long-day crops often maintain an extended 22-hour photoperiod with a two-hour dark phase and cooler nights to accelerate generation turnover and mitigate stress (Watson, 2019). Many major crops, including wheat, barley, canola, chickpea, pea, durum wheat, and oat, are routinely grown under a 22-hour photoperiod as the accepted speed breeding protocol (Watson et al., 2018; González-Barrios et al., 2021). Our results indicate, however, that photoperiod requirements for optimal flowering vary considerably depending on the alleles present at the *PPD-H1* locus. Lines with the *ppd-H1* allele reach a threshold photoperiod at 20 hours, while those with the *Ppd-H1* allele are unresponsive to photoperiods longer than 16 hours—a pattern also observed by Parrado et al. (2023). This suggests that photoperiod length can be fine-tuned to the genetic background of breeding materials: for all genotypes, the widely used 22-hour light regime exceeds what is necessary for accelerated flowering. Based on calculations from Zhang et al. (2017) as described in **Supplementary Data S3**, adjusting photoperiods accordingly could reduce lighting demands in the range of 9%–27%—or 475–1,423.5 kWh per 8 sq. ft. annually—translating to both energy and cost savings across facilities. At current UK commercial electricity rates (approximately £0.22 per kWh; UK Department for Energy Security and Net Zero, 2024), these savings would equate to approximately £392,000 to £130,000 for a 10,000 sq ft. commercial facility for a 16h and a 20h photoperiod, respectively, compared to a 22h photoperiod. Moreover, reducing lighting hours not only lowers direct electricity costs, but also decreases the demand for cooling and ventilation, further enhancing the sustainability and cost-efficiency of breeding facilities.

In addition to the primary objective of refining genotype tailored speed breeding protocols, this study investigates how specific allelic combinations influence two parameters of the photoperiod response model: *photoperiod sensitivity* and *intrinsic earliness*, which are utilized here as proxies to understand the modulation of growing season length in function of climate adaptability. As climate warming alters the growing season in parts of Europe, the adaptive advantage conferred by *ppd-H1* alleles in barley cultivars is likely to diminish, highlighting the need to address this issue in future breeding strategies (Herzig et al., 2018). In addition, the warming temperatures may allow for a shift poleward of the agricultural areas, thereby raising the need to understand how crops respond to extremely long photoperiods. Our analysis in this paper helps define how specific allelic combinations influence photoperiod sensitivity and intrinsic earliness. It indicates that combining *PhyC-e* or *ELF3_Hsp_* alleles in a *ppd-H1* background offers a valuable alternative to using *Ppd-H1* alleles. By facilitating crops to escape heat and drought stress without excessively shortening the growing season and therefore enhancing sink related traits such as final leaf number and leaf size (Digel et al., 2016; Parrado et al., 2023), which are pivotal for setting the growth rate during the critical window of development (Slafer et al., 2023), this strategy could offer a more balanced adaptation to warming climates than relying exclusively on *Ppd-H1*. In addition, the *ppd-H1* allele has been shown to enhance spikelet survival by reducing tip degeneration (Huang and Schnurbusch, 2024) and to promote greater floral primordia survival, which translates into improved spike fertility and potentially higher yield in both *PhyC-e* and *PhyC-i* backgrounds (Parrado et al., 2025). Furthermore, *ELF3_Hsp_* has recently been discovered to contribute to phenotypic and developmental acclimation to elevated temperatures (Zhu et al., 2023). Additionally, by showing variability in flowering time under extremely long photoperiods (18–20 hours), the results demonstrate that specific allele combinations enable intermediate flowering times. These combinations also retain photoperiod sensitivity, providing greater flexibility for breeding climate-resilient varieties. Taken together, these findings support the continued exploration and strategic deployment of *ppd-H1* alleles, especially in combination with alleles such as *PhyC-e* or *ELF3_Hsp_*.

The limitations of this study include the need for further validation of our field adaptation results through additional research. First, within the photoperiod range we tested (≥16 h), we could not empirically model responses at shorter daylengths. Consequently, the 16 h breakpoint for genotypes carrying Ppd-H1 was supported by the integration of prior evidence (Parrado et al., 2023), and our estimates do not fully capture the threshold photoperiod and photoperiod sensitivity below 16 h, where many central and northern European environments experience 13–14 h daylengths during early vegetative development. Second, while expressing development as thermal time to heading helped us account for temperature effects and isolate the photoperiod response, we did not quantify photoperiod × temperature interactions that are equally relevant to field conditions. Third, we concentrated on time to heading and did not measure additional developmental traits that mechanistically link phenology to yield formation, such as final leaf number (FLN), leaf size, and growth rate from terminal spikelet to anthesis, traits that are also influenced by prior tillering stage; incorporating these metrics would enable to directly quantify the effects of the allelic combination proposed in crop models. Finally, future work should couple the photoperiod response model with a temperature model and measure relevant phenology traits and yield components allowing joint estimation of sensitivity to daylength and temperature across developmental phases and improving the applications of such results to field conditions.

## Data Availability

Data and scripts can be found in a GitHub public repository at https://github.com/Nic155/From-photoperiod-threshold-to-photoperiod-sensitivity.git.

## Supplementary material

Supplementary Figure 1 Illustration of the grow tent setup showing how different light regimes were applied across experimental conditions in the photoperiod study.

Supplementary Figure 2 Barplots of thermal time to heading under 16 h and 20 h photoperiods across lines.

Supplementary Table 1 Genotype data from the Infinium iSelect 50k SNP chip of HEB group lines for the seven major flowering time loci. The markers utilized for flowering time loci were sourced from the panel of pre-selected (SNPs) described by Zahn et al. (2023).

Supplementary Table 2 **(A, B)** The significance of differences in thermal time to heading between photoperiod treatments (16 h vs. 20 h and 20 h vs. 22 h) was determined for each genotype by Student’s t-test, with mean ± SD reported and a significance level of p < 0.05.

Supplementary Table 3 **(a)** List of primer pairs used in the resequencing of the *PPDH1* and *ELF3* genomic regions. **(b)** List of primers employed in Sanger sequencing; primers in bold provided acceptable sequences for the haplotype analysis. **(c)** List of primer pairs for gene expression analysis.

Supplementary Table 4 Significant differences in PPD-H1 expression fold-change rates between genotypes of HEB lines group within the same photoperiod condition, quantified via RT-qPCR and assessed using Student’s t-test at different time points.

Supplementary Table 5 Significant differences in FT1 expression fold-change rates between genotypes of HEB lines group within the same photoperiod condition, quantified via RT-qPCR and assessed using Student’s t-test at different time points.

Supplementary Data Sheet 1 Molecular characterization of *ELF3* and *PPD-H1* allelic variation in HEB lines.

Supplementary Data Sheet 2 Overview of the photoperiod response modeling strategy.

Supplementary Data Sheet 3 Energy savings calculations.
