# Supplementary material for: From photoperiod thresholds to photoperiod sensitivity: dual strategies for cost-effective speed breeding and climate-ready barley
Source: Front Plant Sci. 2026 Feb 10;17:1742787. doi: 10.3389/fpls.2026.1742787 (PMC12929436; doi:10.3389/fpls.2026.1742787)
Supplement: Supplementary Figure 1 — Illustration of the grow tent setup showing how different light regimes were applied across experimental conditions in the photoperiod study. [file DataSheet1.docx]

**Supplementary Figures**

**
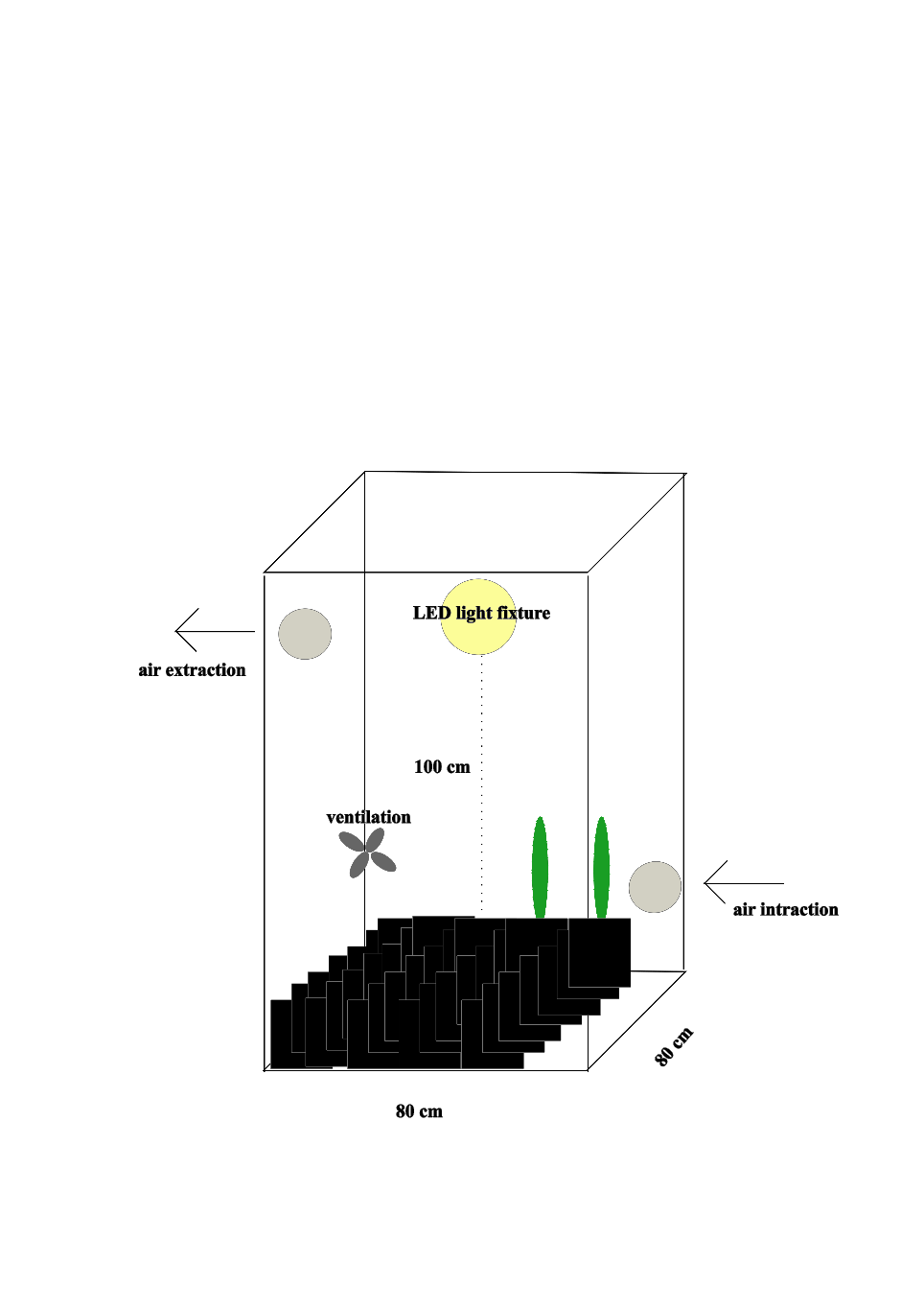
**

**Figure S1**. Illustration of the grow tent setup showing how different light regimes were applied across experimental conditions in the photoperiod study.

**
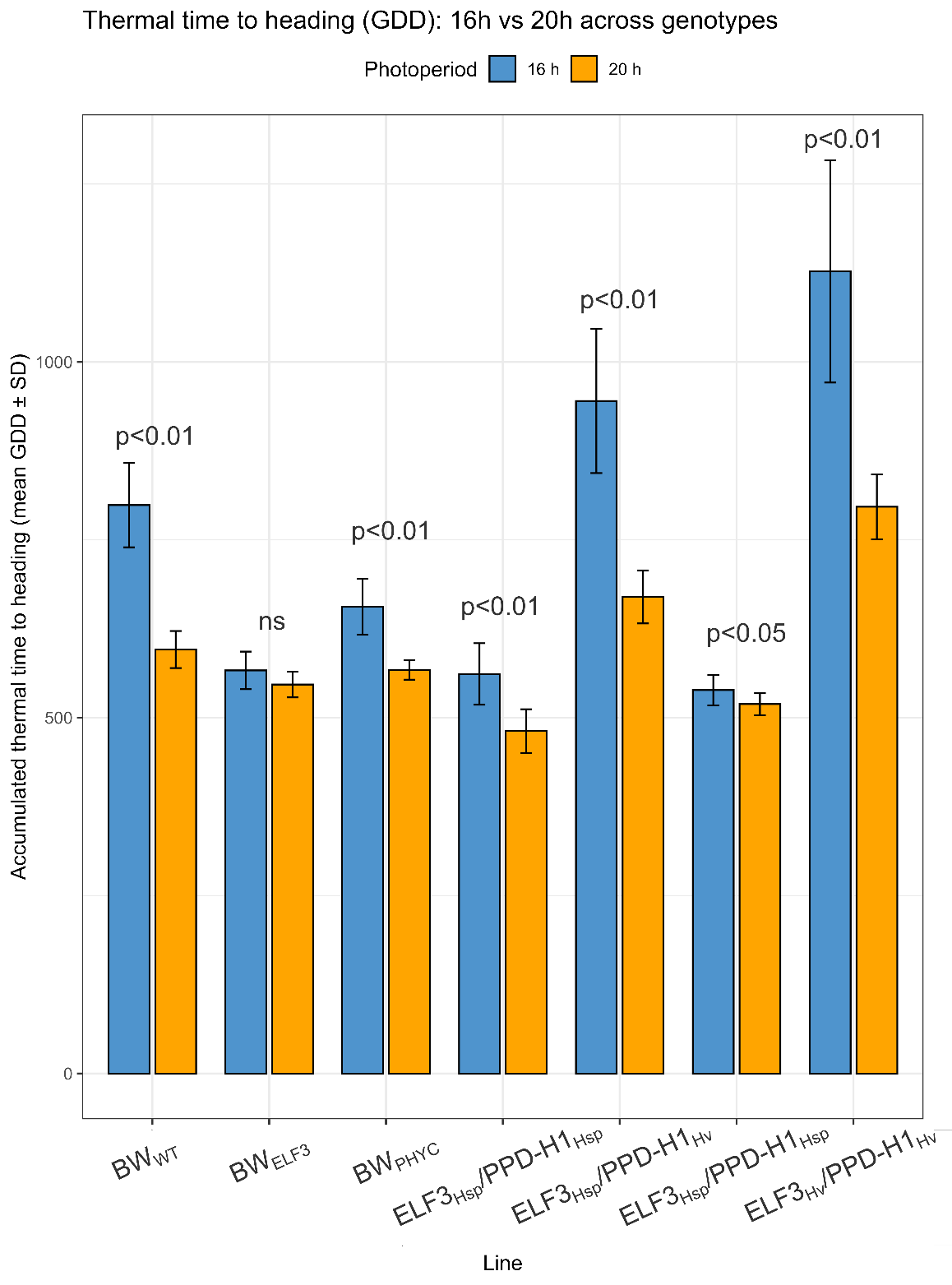
**

**Figure S2**. Barplots of thermal time to heading under 16 h and 20 h photoperiods across lines.

**Supplementary Data**

**Data S1 Graphical genotyping of *ELF3* and *PPD-H1* Allelic Variation in HEB group**

Part of the sequence of *ELF3* and *PPD-H1* (that includes the SNP from Turner et al., (2005)) from Barke, the 4 lines in the HEB lines group and its donor (HID_065) were amplified using Thermo Fischer Scientific’s Phusion Plus PCR Master Mix for ELF3 and Qiagen’s *Taq* PCR Master Mix Kit for *PPD-H1*. Amplicons were purified with Thermo Fischer Scientific’s ExoSAP-IT™ PCR Product Cleanup Reagent and sent for Sanger sequencing to DBS genomics. One fragment for *PPD-H1* and two overlapping fragments from *ELF3* were successfully assembled and then aligned to the reported coding sequences from Barke (Maurer et al., 2015; Zahn et al., 2023). The primers used for amplification and sequencing, along with the PCR amplification conditions are reported in Tables S3a and b. Sequence alignment was performed using Bioedit (Hall, T.A 1999).

To ensure that the RILs used in the HEB family group harboured the different *ELF3* and *PPD-H1* alleles we sequenced these genomic regions, comparing them with Barke and Bowman.

For *ELF3* it wasn’t possible to confidently align the first ~490 bp and the region going from ~ 1100 to ~ 1200 bp. The primers gathered from Zahn et al., (2023) that enabled the sequencing of the region are 5’ - AGCATACTCTGAAGCGCTAATTG – 3’ and 5’- TAGTTCACACGGCAGAGACA -3’. The sequence obtained still cover most of the gene and this allowed for depicting genetic and protein haplotypes harbouring key mutations (Table I). The two lines harbouring *ELF3_Hsp_* (RILs 05_047 and 05_048), selected from marker data, showed the same haplotype with SNPs at positions 516, 1053, 1101, 1830, 1958 and 2005 compared to Barke. Positions 1958 and 2005 give rise to aminoacidic mutations at positions 653 and 669. Whereas Bowman showed genetic mutations at positions 516, 944, 1101, 1569, 2005 and 2093. Positions 2005 and 2093 give rise to aminoacidic mutations at positions 669 and 698, thus sharing with the two RILs that harbour *ELF3_Hsp_* only the modification at position 669.

| *Genotypes* | NUCLEOTIDE SEQUENCE | | | | | | | | | | | | *AMINOACID SEQUENCE* | | | |
| --- | --- | --- | --- | --- | --- | --- | --- | --- | --- | --- | --- | --- | --- | --- | --- | --- |
|  | *ELF3 (50k)* | 516 | 608 | 944 | 1053 | 1101 | 1569 | 1596** | 1830 | 1958 | 2005 | 2093 | 653 | 669* | 698 |  |
| Barke |  | C | C | C | C | G | C | C | G | T | T | T | V | W | L |  |
| 05_019 | *0* | C | C | C | C | G | C | C | G | T | T | T | V | W | L |  |
| 05_040 | *0* | C | C | C | C | G | C | C | G | T | T | T | V | W | L |  |
| 05_047 | *2* | T | C | C | T | A | T | C | A | C | G | T | A | G | L |  |
| 05_048 | *2* | T | C | C | T | A | T | C | A | C | G | T | A | G | L |  |
| Bowman |  | T | C | G | C | A | T | C | G | T | G | C | V | G | P |  |

**Table I**. *ELF3* haplotypes for the HEB group, Barke and Bowman. * Amino acid substitution found by Zahn et al., (2023), ** causal SNP of such substitution.

As for *PPD-H1*, three haplotypes were depicted from the genetic sequences, this is surprising since they all belong to the same HEB family. (Table II) However, the lines selected from marker data for having the wild *PPD-H1* both harbour the SNP from Turner et al., (2005), in position 843, which leads to the mutation in the CCT domain that shortens flowering time, such mutation is also harboured by Bowman (Faure et al., 2012).

| *Genotypes* | *PPD-H1 (50k)* | 84 | 89 | 119 | 254 | 286 | 356 | 357 | 358 | 359 | 360 | 361 | 464 | 483 | 564 | 843* | 876 |
| --- | --- | --- | --- | --- | --- | --- | --- | --- | --- | --- | --- | --- | --- | --- | --- | --- | --- |
| Barke |  | T | C | G | T | C | - | - | - | - | - | - | G | A | G | **T** | A |
| 05_048 | *0* | T | C | G | T | C | - | - | - | - | - | - | G | A | G | **T** | A |
| 05_040 | *0* | T | C | G | T | C | - | - | - | - | - | - | G | A | G | **T** | A |
| 05_047 | *2* | T | C | A | G | C | - | - | - | - | - | - | G | G | A | **G** | G |
| 05_019 | *2* | C | T | A | G | G | A | G | C | - | - | - | A | G | G | **G** | G |
| Bowman |  | C | T | A | T | G | - | - | - | - | - | - | A | G | G | **T** | G |

**Table II.** *PPD-H1* haplotypes for HEB group, in bold, position 843, the SNP from Turner et al (2005) that divides ppd-H1 from Ppd-H1.

**Data S2 Modelling strategy**

Based on the visual assessment of the means and the conceptual models developed by Perez-Gianmarco et al., (2019), three different models were fitted: one for the *PPD-H1_Hv_* lines, one for the *PPD-H1_Hsp_* lines and another for the Bowman group. Tables III and IV summarize the prior knowledge that informed these modeling approaches.

| **Genotype (HEB group)** | **Key Allele(s)** | **Photoperiod Sensitivity in the range 16 – 20** | **Intrinsic Earliness** | **Photoperiod Threshold (h)** |
| --- | --- | --- | --- | --- |
| ELF3_Hv_/PPD-H1_Hv_ | *ELF3_Hv_ (Elf3) + PPD-H1_Hv_ (ppd-H1)* | Yes | High | 20 |
| ELF3_Hsp_/PPD-H1_Hv_ | *ELF3_Hsp_ + PPD-H1_Hv_ (ppd-H1)* | Yes | Lower than above | 20 |
| ELF3_Hv_/PPD-H1_Hsp_ | *ELF3_Hv_ (Elf3) + PPD-H1_Hsp_ (Ppd-H1)* | No | Baseline | 16 |
| ELF3_Hsp_/PPD-H1_Hsp_ | *ELF3_Hsp_ + PPD-H1_Hsp_ (Ppd-H1)* | No | Baseline | 16 |

**Table III.** Photoperiod response and intrinsic earliness in HEB genotypes. Summary of HEB group genotypes, their key allelic combinations, photoperiod sensitivity between 16–20 hours, intrinsic earliness, and the photoperiod threshold at which further increases in daylength no longer accelerate flowering

| **Genotype (Bowman group)** | **Key Allele(s)** | **Photoperiod Sensitivity** | **Intrinsic Earliness** | **Photoperiod Threshold (h)** |
| --- | --- | --- | --- | --- |
| BW_WT_ (wild type) | *Elf3 + Phyc-I + ppd-H1* | Highest sensitivity | Baseline | 20 |
| BW_PHYC_ | *Elf3 + PhyC-e + ppd-H1* | Moderately reduced | Baseline | 20 |
| BWELF3 | *elf3 + Phyc-I + ppd-H1* | No | Baseline | None |

**Table IV**. Photoperiod response characteristics of Bowman near-isogenic lines. Summary of Bowman group genotypes, highlighting key alleles, photoperiod sensitivity, intrinsic earliness (which is equivalent for all lines under non-inductive conditions), and photoperiod threshold. BWELF3 is photoperiod-insensitive.

For the lines carrying the PPD-H1Hv alleles In the HEB group a bi-linear equation was adopted. It describes how the expected thermal time to heading decreases at a specific rate, determined by the photoperiod sensitivity (β) until reaching a threshold photoperiod ( γ), where the thermal time to heading is at its minimum. Beyond this threshold, there is no further acceleration of flowering with increasing photoperiod. This point is identified as the intrinsic earliness (α). Therefore, in the modelling strategy, the photoperiod sensitivity was kept constant, while the intrinsic earliness varied among the lines.

Model for the lines carrying *PPD-H1_Hv_* in the HEB group (1)

$$y_{ij}\sim N(\mu_{ij},\sigma_{y}^{2})$$

$$\mu_{ij}=\left\{ \begin{aligned} \alpha_{j}+\beta\left( x_{ij}-\gamma\right),ifx_{ij}<\gamma\\ \alpha_{j} otherwise \end{aligned} \right.$$

$$\alpha j\sim N(\alpha,\sigma_{\alpha}^{2})$$

Where $y_{ij}$ is the thermal time to heading of observation i of line j, $x_{ij}$ is the photoperiod from observation i of line j, $\mu_{ij}$ is the expected thermal time to heading, $\sigma_{y}^{2}$ is the error variance, $\beta$ is the mean photoperiod sensitivity of both genotypes ELF3_Hsp_/PPD-H1_Hv_ and ELF3_Hv_/PPD-H1_Hv_ , $\gamma$ is the threshold photoperiod (fixed at 20h), $\alpha_{j}$ is the intrinsic earliness of line j, and $\sigma_{\alpha}^{2}$ is the line variance of intrinsic earliness. Such model was conceptualized in Perez-Gianmarco et al., (2019) as “Model H”.

Whereas for the lines carrying the *PPD-H1_Hsp_* alleles a non-hierarchical Bayesian model was chosen based on the assumption that such genotypes have the same intrinsic earliness ($\alpha$) and show no response to photoperiod. Hence a linear model was adopted which did not consider threshold photoperiod and photoperiod sensitivity.

Model for the lines carrying *PPD-H1_Hsp_* in the HEB group (2)

$$y_{ij}\sim N(\mu_{ij},\sigma_{y}^{2})$$

$$\mu_{ij}=\alpha$$

Where $y_{ij}$ is the thermal time to heading of observation I of line j, $x_{ij}$ is the photoperiod from observation i of line j, $\mu_{ij}$ is the expected thermal time to heading, $\sigma_{y}^{2}$ is the error variance, and $\alpha$ is the mean intrinsic earliness of lines ELF3_Hsp_/PPD-H1_Hsp_ and ELF3_Hv_/PPD-H1_Hsp_ *.* This model aligns to what found also in Parrado et al., (2023) and confirmed by our data, where the sensitive *Ppd-H1* allele had reached intrinsic earliness at 16h.

In the Bowman group, a hierarchical Bayesian model considering a bi-linear equation as described for lines carrying *PPD-H1_Hv_* in the HEB group was adopted. Although here, the photoperiod sensitivity ($\beta$) varied among the lines and the intrinsic earliness was stable ($\alpha$).

Model Bowman group (3)

$$y_{ij}\sim N(\mu_{ij},\sigma_{y}^{2})$$

$$\mu_{ij}=\left\{ \begin{aligned} \alpha+\beta_{j}\left( x_{ij}-\gamma\right),ifx_{ij}<\gamma\\ \alpha otherwise \end{aligned} \right.$$

$$\beta j\sim N(\beta,\sigma_{\beta}^{2})$$

Where $y_{ij}$ is the thermal time to heading of observation i of line j, $x_{ij}$ is the photoperiod from observation i of line j, $\mu_{ij}$ is the expected thermal time to heading, $\sigma_{y}^{2}$ is the error variance, $\alpha$ is the mean intrinsic earliness of lines BW_WT_, BW_ELF3_, and BW_PHYC_*,* $\gamma$ is the threshold photoperiod (fixed at 20h), $\beta_{j}$ is the photoperiod sensitivity of line j, and $\sigma_{\beta}^{2}$ is the line variance of photoperiod sensitivity. Such model was conceptualized in Perez-Gianmarco et al., (2019) as “Model E”.

Model E yielded a *R^2^* of 0.78, whereas model H model yielded a *R^2^* of 0.87.

**Data S3 Energy savings calculations**

Methods note: Energy-savings calculation. Assumptions from Zhang et al., (2017):

- LED power density: 650 W per 8 ft² (i.e., 0.65 kW per 8 ft²).
- Operating hours in Zhang et al.: 3,000 h/year (over 15 years → 45,000 h total).
- Total LED electricity per 8 ft² over 15 years: 0.65 kW × 45,000 h = 29,250 kWh (Table 2 in Zhang et al.).
- Annual LED electricity per 8 ft²: 29,250 ÷ 15 = 1,950 kWh/year.

Derivations

- Per‑hour consumption: 1,950 kWh/year ÷ 3,000 h/year = 0.65 kWh per hour of operation → consistent with 0.65 kW installed load.
- If SB rooms run daily (365 d/year), the saving per 8 ft² for cutting 1 h/day is: ΔE(1 h/day) = 0.65 kW × 365 h = 237.25 kWh/year
  - 22→20 h (2 h/day): 2 × 237.25 = 474.5 kWh/year per 8 ft²
  - 22→16 h (6 h/day): 6 × 237.25 = 1,423.5 kWh/year per 8 ft²

Scaling to facility size

- For area A (ft²): multiply by (A ÷ 8). Example for 10,000 ft² (= 1,250 blocks of 8 ft²):
  - 22→20 h: 474.5 × 1,250 ≈ 593,125 kWh/year → ≈£130.000/year at £0.22/kWh
  - 22→16 h: 1,423.5 × 1,250 ≈ 1,779,375 kWh/year → ≈£392.000/year
